# Neuromotor Development of Children Aged 6 and 7 Years Born before the 30th Week Gestation

**DOI:** 10.1155/2018/2820932

**Published:** 2018-05-20

**Authors:** Joanna Majewska, Katarzyna Zajkiewicz, Kamila Wacław-Abdul, Joanna Baran, Daniel Szymczyk

**Affiliations:** ^1^Institute of Physiotherapy, Medical Faculty, University of Rzeszow, Rejtana 16c, 35-959 Rzeszów, Poland; ^2^Centre for Innovative Research in Medical and Natural Sciences, Medical Faculty, University of Rzeszow, Warzywna 1a, 35-310 Rzeszów, Poland

## Abstract

**Introduction:**

The aim of this study was to evaluate and compare the level of neuromotor function and somatic development in 6- and 7-year-old children born before the 30th week gestation with that in full-term children at the same age, as well as the correlation between prematurity and motor development.

**Material and Methods:**

The study group consisted of prematurely born 40 children. Their mean gestational age at birth was 27.8 ± 1.6 weeks (range 24–30 weeks). The control group consisted of 40 healthy children born with normal birth weight (>2500 g). The neuromotor function was assessed using Touwen neurological examination criteria. During the examination, the attention was focused on the hand preference, laterality, synkinesis, and asymmetry. In addition, children's weight, height, and BMI index were measured.

**Results:**

Premature children showed much worse results than full-term ones in hand function (*p* < 0,001). They obtained the best results in paper tearing while crossing the body midline turned out to be the most difficult. Considering the quality of walking, the biggest difficulty for the premature children was to walk backwards along the straight line while during normal walking they showed the best results. The results for the muscle tone subcategory in the study group were also significantly worse than those in control group (*p* < 0,001), as well as the total outcome for the movement coordination and diadochokinesis subcategories (*p* < 0,001).

**Conclusion:**

The nondisabled, prematurely born children have significantly lower average outcomes regarding hand function, quality of walking, muscle tone, coordination, and diadochokinesis at age of six to seven, compared to the full-term peers.

## 1. Introduction

The incidence of preterm delivery has been increasing and the survival rate of preterm children has risen steadily due to advances in obstetric and neonatal intensive care [1]. According to the World Health Organization (WHO), the infants born before 32nd week of pregnancy are considered very preterm (VPT) infants [2]. The WHO estimated that, in 2015, 15 million infants were born before 37 weeks of gestation. Across 184 countries, the rate of preterm birth ranges from 5% to 18%. Preterm infants are being allocated to two categories according to their birth weight: low birth weight (LBW) and very low birth weight (VLBW) when their birth weight ranges from <2500 grams and <1500 grams, respectively [3].

Recent studies show a diminishing prevalence of severe motor disabilities in preterm children, but mild neurodevelopmental impairments remain dominating problems for preschool and school-aged children [4]. Children born prematurely may present delays in the motor [5–8], adaptive [9], cognitive [5, 6, 8, 10], and language [5, 6, 8, 11] domains, even if the deficits in these areas are subtle [5]. These domains are interdependent; that is, each one influences and is influenced by the other.

Motor deficits in coordination, balance, gross and fine motor control, visual spatial, and visual motor integration have been reported in preterm children without CP, but can be more accurately evaluated at a later age [12, 13]. Among other characteristics, parents describe such children as “clumsy,” with decreased hand-eye coordination and motor control challenges. These mild neurodevelopmental impairments tend to persist into later childhood, which might challenge children's successful participation in everyday life both at school and at home [14, 15].

Although long-term developmental changes in preterm children are well described in the literature, the occurrence of these difficulties at preschool age is less documented. Neither the degree of prematurity nor early cognitive testing predicts which children within nondisabled, preterm groups will have poorer functional performance and will require extra services [16]. Therefore, further investigation to obtain a more in-depth understanding of the impairments and activity limitations among nondisabled extremely preterm or ELBW children at preschool age is required. The findings will provide useful information to assist in the development of strategies to provide better results for this population of children.

## 2. Purpose

The aim of this study was to evaluate and compare the level of neuromotor function and somatic development in 6- and 7-year-old children born before the 30th week gestation with that in full-term children at the same age, as well as the correlations between prematurity and motor development.

## 3. Material and Methods

Ethical approval for the study was granted by the Ethics Committee of the University of Rzeszow. Written formal consent was obtained from the parents of all subjects who participated in this study.

### 3.1. Participants

Data for this study was collected between August 2007 and July 2009. The study group consisted of prematurely born 40 children (19 females, 21 males), whose personal data were acquired from the database of the Neonatology Department at the County Hospital No. 2 in Rzeszów, Poland. Their mean gestational age at birth was 27.8 ± 1.6 weeks (range 24–30 weeks). Mean birth weight was 1124 g (range 570 g–1300 g). Mean chronological age was 6 years and 8 months (range from 5 years and 9 months to 7 years and 4 months). The authors excluded children with major congenital malformations, genetic chromosomal abnormalities, metabolic disorders, cerebral palsy (CP) that interfered with locomotion, congenital infections, sign of encephalopathy or seizures during their neonatal course, and retinopathy of prematurity greater than stage 2, because it had been assumed that these infants would have already developed poor neurodevelopmental disorders when compared with healthy children. Children with a visual impairment not corrected by wearing corrective lenses and those with a hearing impairment not corrected by hearing aids were also excluded. The early medical status of preterm infants was extracted from medical records. Then parents of eligible children were contacted to inquire whether they wished to receive further information and appropriate consent forms to complete.

The control group consisted of 40 healthy children (20 females, 20 males) born with normal birth weight (>2500 g), the pupils of primary schools in Rzeszów who were born between February 2002 and January 2003. First, requests to the headmasters of these schools were submitted in order to obtain the school's permission for participation in the research. Next, the children received a letter to their parents, which explained the purpose of the study and requested their consent regarding the children participation in this study. Children with a history of admission to neonatal intensive care unit, gestation of <37 or >42 weeks, infants born from multiple pregnancies, and those with musculoskeletal, neurological, genetic, and other disorders that could negatively influence motor development were excluded from the control group. The proper developmental status of children in the control group had been confirmed by paediatricians based on previous medical periodic examinations. Data concerning children's birth and neonatal status were provided by their parents. Children whose parents signed the formal written consent confirming their participation in the study were evaluated.

### 3.2. Procedure

Standardized and age-specific assessment, according to Touwen neurological examination criteria for children with minor neurological dysfunction (MND), was used in this study [17]. The Touwen neurological examination is free of charge, well-known, and commonly used assessment method, which is specially designed for the evaluation of the following minor neurological dysfunction in children: mild abnormalities in muscle tone (clinical test by passive movement), diadochokinesis, quality of walking, posture, mild problems with coordination, and hand function.

Modified, performance based assessment criteria were applied for the evaluation of all the tests in our study, considering all subcategories (hand function, quality of walking, muscle tone, movement coordination, and diadochokinesis). Each test was scored using 4-point scale, from 0 to 3 (0 = lowest possible score up to 3 = best, optimal score). Then the numeric scores were categorized as a “lack of skills,” weak, medium and “optimal,” respectively. Additionally, total outcome in each subcategory was calculated, as a percentage of optimal score, in the following manner: each child score described as “lack of skills” was graded as 0% while that described as “optimal” was graded as 100%. Overall outcome, as a sum of the average scores in each subcategories, divided by the number of subcategories, was also calculated. During the examination, the attention was also focused on the hand preference, laterality, synkinesis, and asymmetry. In addition, children's weight (kilograms), height (centimeters), and the BMI index (kg∖m2) were measured.

All assessments were completed by the same experienced physiotherapist who was trained in testing protocol and who had no previous access to information regarding the birth status and medical history of the subjects. Each child was evaluated under the same environmental conditions and completed all the testing in one day. Each assessment took approximately one hour and took place in the County Hospital No. 2 in Rzeszów.

### 3.3. Statistical Analysis

All calculations and statistical analyses were performed using STATISTICA ver. 10.0 (StatSoft, Poland). Statistical significance level was assumed at *p* ≤ 0.05. The Shapiro-Wilk test was used for the evaluation of normal data distribution. Basic descriptive statistical analyses were conducted for all variables. Adequate parametric or nonparametric statistical tests, depending on the data type and distribution, were used for the comparison of the results between the groups. Independent *t*-test was used for normally distributed data. The Chi-Square test was used to determine statistically significant differences for categorical measures while Spearman's rank correlation coefficient was used to measure the strength and direction of correlation between two sets of data.

## 4. Results

There were no statistically significant differences between the level of somatic development of children in both groups (Table 1).

There are statistically significant differences regarding hand preferences between the groups; in the study group there were less right handed children and more both handed. Sixty-eight percent of the children in study group were right handed compared to 88% in the control group. Moreover, the fact that 25% of the subjects in the group of preterm children were both handed while in control group none of children presented both hand preferences seems to be very interesting (Table 2).

The differences of the results of all tests in hand function subcategory between the study and control group were statistically significant. Premature children showed worse results than those born full term. Considering hand function, premature children obtained the best results in paper tearing while crossing the body midline turned out to be the most difficult (Table 3).

In the quality of walking subcategory, the biggest difficulty for premature children was to walk backwards along straight line while assessment of normal walking provided the best results (Table 4).

The results of all tests in muscle tone subcategory were significantly worse in the study group, compared to the control group (Table 5).

In all tests evaluating movement coordination, the children from study group obtained significantly worse results than the children from control group (Table 6).

In diadochokinesis subcategory the biggest difficulty for premature children were observed in reaching for the ears with crossed arms test and finger-opposition test (Table 7).

Comparison of the total outcomes for each of the functional subcategories in our study revealed significant differences between preterm children and their full-term peers (*p* < 0,001). The difference in overall outcome (average level of the results considering all subcategories) was also statistically significant (*p* < 0,001) (Table 8).

The correlations between gestation time and the results of each test used in our study, as well as total outcome for the subcategory, were also analysed. The degree of prematurity had statistically significant influence only on the results of three tests in the hand function subcategory, while the strength of those correlations was moderate (Table 9).

## 5. Discussion

Both technological advances in neonatology over the last few decades and increased survival of preterm infants have made it important to consider the long-term outcomes concerning their developmental status. Children born prematurely may present developmental delays even in the absence of severe neurological disorders [6–8, 18–20]. Many researchers are interested in investigating the preterm children population without major neurological impairments because milder functional problems are often not being diagnosed until these children reach their school age [21]. These subtle movement dysfunctions do not occur due to known physical disorders, such as cerebral palsy, hemiplegia, or muscular dystrophy. It is estimated that 40–70% of children born prematurely are showing minor disabilities such as mild motor problems and poor adaptive behaviors during preschool and school years [9]. School children born with extreme prematurity, without any significant neurological problem or developmental impairment presented worse performance in sensorimotor and visuospatial competencies, as well as in attention and executive function when compared with children born at term [10]. Therefore, it is very important to identify potential neurodevelopmental impairments at younger age in order to introduce more efficient consultation and timely intervention.

The objective of this study was to determine whether children born before 30 weeks of pregnancy, who seemed to be free of serious neurological disorders, would develop similar growth patterns and neurodevelopmental performance at preschool age, compared with their full-term peers.

One of the diagnostic methods, suitable for children with motor development disorders, which was used in this study, was Touwen neurological examination. This is a standardized and age-specific assessment tool that focuses on the presence of minor neurological dysfunction.

The results of the recent study demonstrate that a significant amount of apparently normal preterm children had worse motor and functional performance at preschool age than their full-term peers [17].

In the scientific studies concerning motor development of preterm children, the incidence of motor impairment is reported to vary from 9.5 to 51% [22–28]. These children may also exhibit other learning difficulties. Several studies have attempted to assess if motor impairments are being associated with learning impairments in preterm children. Motor impairments are definitely associated with intellectual estimations of both the visual and verbal domains [22]. When children with motor impairments are compared with their full-term peers, differences are seen in a wide variety of performed intellectual and academic tasks [23, 24].

Analysis of the relationship between the duration of pregnancy and the results of the individual tests showed that the degree of prematurity affects, in a statistically significant way, only the results of the three tests in the hand function subcategory. However, the strength of these correlations was moderate. It has been reported by de Kieviet et al. that gestational age is related to delayed motor performance occurring during early developmental period in children under 5 years of age [29].

Defining mild neuromotor impairments at preschool age, as a valid marker of long-term impairments, allows proactive support and prospective allocation of resources to be directed to those who are most likely to struggle with future problems and limitations in personal, social, and academic life.

## 6. Conclusion

The results of our study indicate that the nondisabled, preterm children have lower average outcomes concerning hand function, quality of walking, muscle tone, coordination, and diadochokinesis at the age of six to seven, compared to their full-term peers. It seems that, with time, these particular children may be prone to emerging problems in the future. Therefore, ongoing screening of these children seems to be essential. Involvement of nondisabled, preterm children in appropriate intervention programs may facilitate their optimal development, maintain adequate motor performance, and minimize the development of long-term impairments.

## Figures and Tables

**Table 1:** Somatic development of children.

	Study group					Control group					*p*
	$\bar{x}$	Me	Min	Max	s	$\bar{x}$	Me	Min	Max	s	
Body height	120,9	121,0	104	133	6,2	120,4	120,0	112	134	5,1	0,668
Body mass	22,8	24,0	14	30	4,0	22,9	22,0	18	32	3,7	0,843
BMI	15,5	16,0	11,8	19,0	2,0	15,7	15,6	12,3	22,2	1,9	0,527

Max: maximum value; Me: median; Min: minimal value; *p*: test probability value; s: standard deviation; $\bar{x}$: mean.

**Table 2:** Hand preferences in study and control group.

Hand preference (*p* = 0,003)	Group		Total
	Study	Control	
Right handed	27 (68%)	35 (88%)	62
Left handed	3 (8%)	5 (13%)	8
Both handed	10 (25%)	0 (0%)	10
*Total *	40	40	80

*p*: test probability value.

**Table 3:** Hand function.

Hand function	Study Group				Control Group				*p*
	Optimal	Medium	Weak	Lack of skills	Optimal	Medium	Weak	Lack of skills	
Copying a picture									
*N*	13	16	7	4	34	6	0	0	**<0,001**
%	33%	40%	18%	10%	85%	15%	0%	0%	
Drawing a picture of a doll									
*N*	10	12	16	2	33	7	0	0	**<0,001**
%	25%	30%	40%	5%	83%	18%	0%	0%	
Tying shoelaces									
*N*	17	8	7	8	31	1	3	3	**0,004**
%	43%	20%	18%	20%	82%	3%	8%	8%	
Writing their name									
*N*	15	10	8	6	33	7	0	0	**<0,001**
%	38%	26%	21%	15%	83%	18%	0%	0%	
Cutting a piece of paper with a pair of scissors									
*N*	16	19	3	2	38	1	0	0	**<0,001**
%	40%	48%	8%	5%	97%	3%	0%	0%	
Tearing a piece of paper									
*N*	25	12	1	2	39	1	0	0	**<0,001**
%	63%	30%	3%	5%	98%	3%	0%	0%	
Putting matches in a box									
*N*	9	22	7	2	39	1	0	0	**<0,001**
%	23%	55%	18%	5%	98%	3%	0%	0%	
Crossing the body midline									
*N*	6	8	23	3	38	2	0	0	**<0,001**
%	15%	20%	57%	8%	95%	5%	0%	0%	

%: percent of tested subjects; *N*: number of tested subjects; *p*: test probability value.

**Table 4:** Quality of walking.

Quality of walking	Study group				Control group				*p*
	Optimal	Medium	Weak	Lack of skills	Optimal	Medium	Weak	Lack of skills	
Quality of normal walking									
*N*	20	14	4	2	40	0	0	0	**<0,001**
%	50%	35%	10%	5%	100%	0%	0%	0%	
Walking on tiptoes									
*N*	1	20	16	3	36	4	0	0	**<0,001**
%	3%	50%	40%	8%	90%	10%	0%	0%	
Walking on heels									
*N*	12	17	6	5	40	0	0	0	**<0,001**
%	30%	43%	15%	13%	100%	0%	0%	0%	
Walking along a straight line									
*N*	9	21	5	4	40	0	0	0	**<0,001**
%	23%	54%	13%	10%	100%	0%	0%	0%	
Walking backward along a straight line									
*N*	0	6	27	6	33	7	0	0	**<0,001**
%	0%	15%	69%	15%	83%	18%	0%	0%	
Quality of running									
*N*	8	20	7	4	39	0	0	0	**<0,001**
%	21%	51%	18%	10%	100%	0%	0%	0%	

%: percent of tested subjects; *N*: number of tested subjects; *p*: test probability value.

**Table 5:** Muscle tone.

Muscle tone	Study group				Control Group				*p*
	Optimal	Medium	Weak	Lack of skills	Optimal	Medium	Weak	Lack of skills	
Manipulation of the right and left shoulders									
*N*	5	28	5	2	39	1	0	0	**<0,001**
%	13%	70%	13%	5%	98%	3%	0%	0%	
Manipulation of the right and left hips with extended knees									
*N*	3	24	10	3	37	3	0	0	**<0,001**
%	8%	60%	25%	8%	93%	8%	0%	0%	
Manipulation of the right and left ankles (dorsal flexion)									
*N*	6	19	12	3	35	5	0	0	**<0,001**
%	15%	48%	30%	8%	88%	13%	0%	0%	
Ability to reach the toes with the fingers while standing with extended knees									
*N*	6	15	10	9	33	7	0	0	**<0,001**
%	15%	38%	25%	23%	83%	18%	0%	0%	
Ability to rotate the trunk while sitting									
*N*	14	20	3	3	39	0	0	0	**<0,001**
%	35%	50%	8%	8%	100%	0%	0%	0%	
Ability to squat down with the heels on the floor									
*N*	5	18	12	5	35	5	0	0	**<0,001**
%	13%	45%	30%	13%	88%	13%	0%	0%	

%: percent of tested subjects; *N*: number of tested subjects; *p*: test probability value.

**Table 6:** Movement coordination.

Movement coordination	Study group				Control group				*p*
	Optimal	Medium	Weak	Lack of skills	Optimal	Medium	Weak	Lack of skills	
Catching a ball									
*N*	28	10	0	2	40	0	0	0	**<0,001**
%	70%	25%	0%	5%	100%	0%	0%	0%	
Skipping gait (light hops on each foot)									
*N*	6	25	6	3	40	0	0	0	**<0,001**
%	15%	63%	15%	8%	100%	0%	0%	0%	
Leaping like a frog									
*N*	9	19	7	4	37	3	0	0	**<0,001**
%	23%	49%	18%	10%	93%	8%	0%	0%	
Slow and fast pronation/supination with arms extended in front									
*N*	9	19	10	2	40	0	0	0	**<0,001**
%	23%	48%	25%	5%	100%	0%	0%	0%	
Flexion and extension of wrists									
*N*	13	14	9	4	40	0	0	0	**<0,001**
%	33%	35%	23%	10%	100%	0%	0%	0%	
Sitting up from supine without the help of the arms									
*N*	3	13	18	6	32	7	0	0	**<0,001**
%	8%	33%	45%	15%	82%	18%	0%	0%	
Hopping on the right or left leg									
*N*	5	24	7	3	39	0	0	0	**<0,001**
%	13%	62%	18%	8%	100%	0%	0%	0%	
Standing on right and left leg (eyes open)									
*N*	10	18	8	3	39	0	0	0	**<0,001**
%	26%	46%	21%	8%	100%	0%	0%	0%	
Standing on right and left leg (eyes closed)									
*N*	1	9	24	6	35	4	0	0	**<0,001**
%	3%	23%	60%	15%	90%	10%	0%	0%	
Knee-heel test in supine with the eyes open and closed									
*N*	3	18	14	4	36	2	0	0	**<0,001**
%	8%	46%	36%	10%	95%	5%	0%	0%	

%: percent of tested subjects; *N*: number of tested subjects; *p*: test probability value.

**Table 7:** Diadochokinesis with closed eyes.

Diadochokinesis with closed eyes	Study group				Control group				*p*
	Optimal	Medium	Weak	Lack of skills	Optimal	Medium	Weak	Lack of skills	
Right-left arm pronation and supination with fast alternating movements									
*N*	5	22	10	2	39	1	0	0	**<0,001**
%	13%	56%	26%	5%	98%	3%	0%	0%	
Finger-nose test									
*N*	14	21	2	3	39	1	0	0	**<0,001**
%	35%	53%	5%	8%	98%	3%	0%	0%	
Reaching for the ears with crossed arms									
*N*	1	12	23	3	35	5	0	0	**<0,001**
%	3%	31%	59%	8%	88%	13%	0%	0%	
Finger-finger test									
*N*	13	18	6	3	40	0	0	0	**<0,001**
%	33%	45%	15%	8%	100%	0%	0%	0%	
Finger-opposition test									
*N*	2	10	25	3	38	2	0	0	**<0,001**
%	5%	25%	63%	8%	95%	5%	0%	0%	

%: percent of tested subjects; *N*: number of tested subjects; *p*: test probability value.

**Table 8:** Subcategory total outcome and overall outcome results: study group versus control group.

Subcategory	Study group					Control group					*p*
	$\bar{x}$	Me	Min	Max	s	$\bar{x}$	Me	Min	Max	s	
Hand function	65%	69%	0%	100%	24%	96%	100%	75%	100%	7%	**<0,001**
Quality of walking	58%	61%	0%	94%	23%	98%	100%	89%	100%	3%	**<0,001**
Passive muscle tone	58%	61%	0%	94%	18%	97%	100%	83%	100%	5%	**<0,001**
Movement coordination	59%	62%	0%	90%	21%	99%	100%	90%	100%	3%	**<0,001**
Diadochokinesis	57%	60%	0%	92%	19%	99%	100%	87%	100%	3%	**<0,001**
Overall outcome	60%	63%	0%	90%	19%	98%	99%	88%	100%	3%	**<0,001**

Max: maximum value; Me: median; M∗in: minimal value; *p*: test probability value; s: standard deviation; $\bar{x}$: mean.

**Table 9:** Evaluation of correlation with gestation time.

Hand function	Correlation with gestation time	
	*R*	*p*
Copying a picture	0,46	**0,003**
Drawing a picture of a doll	0,38	**0,014**
Tying shoelaces	0,24	0,143
Writing their name	0,37	**0,021**
Cutting a piece of paper with a pair of scissors	−0,02	0,890
Tearing a piece of paper	0,16	0,333
Putting matches in a box	0,09	0,573
Crossing the body midline	0,20	0,205
Total outcome	0,33	**0,036**

Quality of walking		
Quality of normal walking	−0,01	0,953
Walking on tiptoes	0,07	0,665
Walking on heels	0,04	0,820
Walking along a straight line	−0,04	0,813
Walking backward along a straight line	0,03	0,870
Quality of running	0,01	0,959
Total outcome	−0,01	0,953

Muscle tone		
Manipulation of the right and left shoulders	0,25	0,116
Manipulation of the right and left hips with extended knees	0,22	0,166
Manipulation of the right and left ankles (dorsiflexion)	0,14	0,375
Ability to reach the toes with the fingers while standing with extended knees	−0,01	0,963
Ability to rotate the trunk while sitting	0,00	0,987
Ability to squat down with the heels on the floor	0,03	0,856
Total outcome	0,18	0,267

Movement coordination		
Catching a ball	0,04	0,799
Skipping gait (light hops on each foot)	0,14	0,402
Leaping like a frog	0,11	0,496
Slow and fast pronation/supination with arms extended in front	0,01	0,973
Flexion and extension of wrists	−0,07	0,648
Sitting up from supine without the help of the arms	−0,01	0,944
Hopping on the right or left leg	0,23	0,155
Standing on right and left leg (eyes open)	0,26	0,110
Standing on right and left leg (eyes closed)	0,04	0,783
Knee-heel test in supine with the eyes open and closed	−0,05	0,745
Total outcome	0,04	0,790

Diadochokinesis with closed eyes		
Right-left arm pronation and supination with fast alternating movements	0,08	0,627
Finger-nose test	0,03	0,870
Reaching for the ears with crossed arms	−0,17	0,313
Finger-finger test	0,24	0,128
Finger-opposition test	0,28	0,075
Total outcome	0,04	0,808

*R*: correlation; *p*: test probability value.
